# Putative Horizontally Acquired Genes, Highly Transcribed during Yersinia pestis Flea Infection, Are Induced by Hyperosmotic Stress and Function in Aromatic Amino Acid Metabolism

**DOI:** 10.1128/JB.00733-19

**Published:** 2020-05-11

**Authors:** Luary C. Martínez-Chavarría, Janelle Sagawa, Jessica Irons, Angela K. Hinz, Athena Lemon, Telmo Graça, Diana M. Downs, Viveka Vadyvaloo

**Affiliations:** a Paul G. Allen School for Global Animal Health, Washington State University, Pullman, Washington, USA; b Departamento de Patología, Facultad de Medicina Veterinaria y Zootecnia, Universidad Nacional Autónoma de México, Mexico City, Mexico; c Department of Microbiology, University of Georgia, Athens, Georgia, USA; d Department of Chemistry and Biochemistry, Gonzaga University, Spokane, Washington, USA; Geisel School of Medicine at Dartmouth

**Keywords:** *Yersinia pestis*, flea infection, hyperosmolarity stress

## Abstract

While alternating between insects and mammals during its life cycle, Yersinia pestis, the flea-transmitted bacterium that causes plague, regulates its gene expression appropriately to adapt to these two physiologically disparate host environments. In fleas competent to transmit Y. pestis, low-GC-content genes *y3555*, *y3551,* and *y3550* are highly transcribed, suggesting that these genes have a highly prioritized role in flea infection. Here, we demonstrate that *y3555*, *y3551,* and *y3550* are transcribed as part of a single polycistronic mRNA comprising the *y3555, y3554, y3553*, *y355x, y3551*, and *y3550* genes. Additionally, *y355x-y3551-y3550* compose another operon, while *y3550* can be also transcribed as a monocistronic mRNA. The expression of these genes is induced by hyperosmotic salinity stress, which serves as an explicit environmental stimulus that initiates transcriptional activity from the predicted *y3550* promoter. Y3555 has homology to pyridoxal 5′-phosphate (PLP)-dependent aromatic aminotransferases, while Y3550 and Y3551 are homologous to the Rid protein superfamily (YjgF/YER057c/UK114) members that forestall damage caused by reactive intermediates formed during PLP-dependent enzymatic activity. We demonstrate that *y3551* specifically encodes an archetypal RidA protein with 2-aminoacrylate deaminase activity but Y3550 lacks Rid deaminase function. Heterologous expression of *y3555* generates a critical aspartate requirement in a Salmonella enterica *aspC* mutant, while its *in vitro* expression, and specifically its heterologous coexpression with *y3550,* enhances the growth rate of an Escherichia coli Δ*aspC* Δ*tyrB* mutant in a defined minimal amino acid-supplemented medium. Our data suggest that the *y3555, y3551*, and *y3550* genes operate cooperatively to optimize aromatic amino acid metabolism and are induced under conditions of hyperosmotic salinity stress.

**IMPORTANCE** Distinct gene repertoires are expressed during Y. pestis infection of its flea and mammalian hosts. The functions of many of these genes remain predicted or unknown, necessitating their characterization, as this may provide a better understanding of Y. pestis specialized biological adaptations to the discrete environments of its two hosts. This study provides functional context to adjacently clustered horizontally acquired genes predominantly expressed in the flea host by deciphering their fundamental processes with regard to (i) transcriptional organization, (ii) transcription activation signals, and (iii) biochemical function. Our data support a role for these genes in osmoadaptation and aromatic amino acid metabolism, highlighting these as preferential processes by which Y. pestis gene expression is modulated during flea infection.

## Abstract (precis)

Distinct gene repertoires are expressed during Y. pestis infection of its flea and mammalian hosts. The functions of many of these genes remain predicted or unknown, necessitating their characterization, as this may provide a better understanding of Y. pestis specialized biological adaptations to the discrete environments of its two hosts. This study provides functional context to adjacently clustered horizontally acquired genes predominantly expressed in the flea host by deciphering their fundamental processes with regard to (i) transcriptional organization, (ii) transcription activation signals, and (iii) biochemical function. Our data support a role for these genes in osmoadaptation and aromatic amino acid metabolism, highlighting these as preferential processes by which Y. pestis gene expression is modulated during flea infection.

## INTRODUCTION

Plague, caused by Yersinia pestis, is a severe zoonotic disease infamous for having caused the death of millions of people in three major pandemics. This disease primarily affects rodents which become infected mainly through blood feeding by rodent-associated infected fleas. Transmission by fleas is a recent adaptation that has likely evolved in the last 1,500 to 20,000 years during the transformation of Y. pestis from its clonal ancestor, Yersinia pseudotuberculosis, which is transmitted by the fecal-oral route and causes a mild enteric self-resolving illness (1–3). Central to the biological mechanism of transmission via flea bite is Y. pestis biofilm blockage of the flea foregut proventriculus (4–6).

After acquisition from the blood of a highly bacteremic host, Y. pestis must adapt to the insect gut environment to multiply and form biofilm-mediated blockage by 7 days postinfection (7). The various physicochemical stresses Y. pestis encounters in the flea gut, such as low pH, reactive oxygen species (ROS), hyperosmolarity, nutrient limitation, and the immune response, are slowly being uncovered, along with how Y. pestis overcomes such stresses (8, 9). For example, reactive oxygen species (ROS) have been demonstrated to limit Y. pestis survival during early flea infection (10), but this oxidative stress can be counteracted by the modulation of antioxidant defenses by the Y. pestis OxyR transcriptional regulator (10). The flea gut was determined as a mildly acidic environment with a pH of ∼6.5 to 6.8 (11). Implicated in adapting to this mild acidic condition is the PhoP transcriptional regulator (12) required to maintain optimal blockage and competitive fitness in fleas and that modulates the transcription of genes required for acid stress (12–14).

Moreover, hyperosmotic stress conditions occur in the flea gut, as its content is measured to be ∼500 mOsm, much greater than that of mouse blood at 320 mOsm (11). However, genes of Y. pestis belonging to pathways involved in alleviating osmotic stress are robustly expressed during flea infection (15–17). Examples of these are (i) genes belonging to the uptake and catabolic pathways of the l-Glu amino acid group (His, Glu, Pro, and Arg) which encodes proteins that generate the osmoprotectant glutamate (17), and (ii) the *betT* and *proVWX* genes of the synthesis and transport system operons for the osmoprotectant glycine-betaine, respectively (15). In addition, the two-component signal transduction regulatory system OmpR-EnvZ that is involved in sensing and mediating adaptation to osmolarity and pH stresses in many bacterial species (18–21), including Y. pestis (22–24), is shown to be required for efficient flea blockage (11).

To further understand the flea host-specific response of Y. pestis, we turned our attention to a cluster of genes (*y3555*, *y3551,* and *y3550*) with lower GC content (39.8%) than that typically noted for Y. pestis (48%) genes and that ranked in the top 150 most highly expressed genes in the flea (17). Close inspection of the chromosomal context of *y3555*, *y3551,* and *y3550* revealed that they are located adjacent to three other genes (*y3554*, *y3553*, and *y355x*) having a similarly low GC content. The *y3554*, *y3553*, and *y335x* genes showed robust expression in the flea gut (9, 17). The *y355x* gene is not annotated as such in the Y. pestis KIM10^+^ genome, but it is 100% identical to ypo0625 of the Y. pestis CO92 genome, the reference to which the microarrays were developed for Y. pestis flea gut infection transcriptomics studies (12, 17). All six of these low-GC-content genes were either poorly expressed or not detected under mammalian conditions. Genes sharing 99 to 100% identity to *y3555*, *y3554*, *y3553*, *y355x*, *y3551,* and *y3550* are present in Y. pseudotuberculosis. Because genetic loss is thought to drive clonal evolution enabling the emergence of Y. pestis and its flea-borne transmissibility (25–28), we reasoned that evolutionary conservation of the encoded traits of these six genes is likely advantageous for growth and survival within new environmental niches like the flea gut. This study therefore aimed to understand the transcriptional organization and activation, as well as the roles of the *y3555*, *y3554*, *y3553*, *y335x*, *y3551,* and *y3550* genes, in flea colonization, with a specific emphasis on the biochemical roles of the most highly expressed genes, *y3555*, *y3550,* and *y3551*. Our data reveal that while these preferentially expressed genes that have been evolutionarily maintained are not essential for flea colonization in the context of our experimental laboratory flea model of Y. pestis infection, they are nonetheless coordinately induced during hyperosmotic stress and contribute to aromatic amino acid metabolism, and *y3555* confers enhanced growth fitness traits to Escherichia coli and Y. pestis *in vitro*.

## RESULTS

### *In silico* analysis of the genetic context and predicted functions of Y. pestis KIM10^+^ genes *y3555*, *y3554*, *y3553*, *y355x*, *y3551*, and *y3550*.

**FIG 1:**
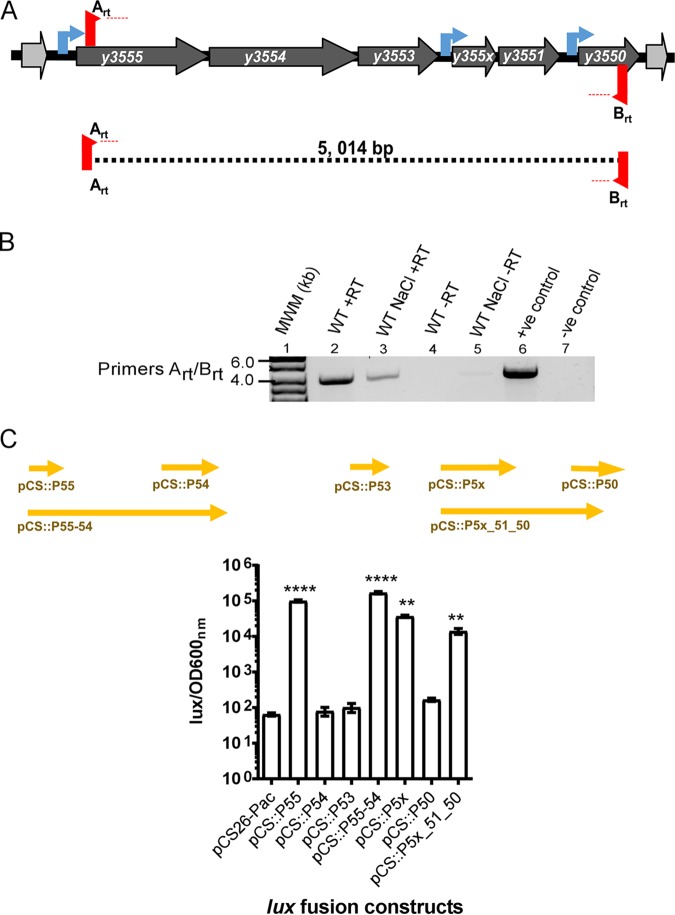
The low-GC-content gene cluster is transcribed as a single polycistronic mRNA. Six genes compose the low-GC-content gene cluster. (A) The three bioinformatically predicted transcriptional units are marked by the presence of predicted promoters upstream of genes *y3555*, *y355x,* and *y3550* (blue arrows). (B) Reverse transcription-PCR (RT-PCR) analysis of regions (indicated by dashed lines) spanning *y3555* to *y3550*. RT-PCRs were performed with reverse transcriptase and DNA polymerase mix (lanes 2 and 3) or only with DNA polymerase (lanes 4 to 7). PCRs in the presence of Y. pestis KIM6^+^ chromosomal DNA (lane 6) or absence of template (lane 7) with the same set of primers were performed as controls. MWM, molecular weight marker; +ve, positive; -ve, negative. (C) The activity of transcriptional fusion plasmids pCS::P55, pCS::P54, pCS::P53, pCS::P55_54, pCS::P5x, pCS::P50, and pCS::P5x_51_50, carrying the transcriptional fusions of the luciferase (*lux*) genes and predicted promoter regions (arrows) representing predicted regulatory regions of *y3555*, *y3554*, *y3553*, *y3555-y3554*, *y355x*, *y3550,* and *y355x-y3551-y3550*, respectively, as well as the low-copy-number empty vector pCS26-*Pac*. Error bars represent mean (±standard deviation [SD]) of the results from three independent experiments. Significance determined by one-way analysis of variance (ANOVA) and a Dunnett’s posttest of *lux*/OD values of transcriptional fusion constructs compared to the empty vector is indicated: ****, *P* < 0.0001, and **, *P* < 0.01.

The lower GC contents of the gene cluster comprising *y3555* (3,935,132 and 3,936,364), *y3554* (3,933,688 and 3,935,139), *y3553* (3,933,238 and 3,933,684), *y355x* (3,932,478 and 3,933,068), *y3551* (3,932,082 and 3,932,462), and *y3550* (3,931,393 and 3,931,797) suggest their acquisition by horizontal gene transfer. *In silico* analysis using FGENESB predicts that the continuous region of this genomic island comprises three transcriptional units, with *y3555-y3554-y3553* and *y355x-y3551* constituting two separate operons and *y3550* representing a monocistronic transcriptional unit. Additionally, BPROM identifies distinct promoter sequences upstream of the genes *y3555*, *y355x,* and *y3550* (Fig. 1A). BLAST searches were used to identify homologous proteins and conserved functional domains of genes comprising the genomic island.

The *y3555* gene product exhibits homology to uncharacterized pyridoxal 5′-phosphate (PLP)-dependent transaminases, including aromatic and aspartate aminotransferases encoded in some bacteria. These PLP-dependent aminotransferases typically catalyze the interconversion of an aromatic amino acid and α-ketoglutarate to oxaloacetate and glutamate. The Y. pestis KIM10^+^ genome encodes two PLP-dependent aromatic aminotransferases, encoded by *y2760* (*aspC*) and *y0579* (*tyrB*).

The *y3554* gene encodes a putative Na^+^/H^+^ antiporter (NhaC type), and there are two other antiporters (NhaA and NhaB) in the reported genome sequence of Y. pestis. A primary role for Na^+^/H^+^ cation exchange antiporters (29–31) is to extrude Na ions to counterbalance Na^+^ toxicity, thereby protecting against hyperosmotic saline stress. The NhaA and NhaB antiporters are essential for Y. pestis virulence in a mouse model of septicemic plague (31, 32), where they are thought to function in protecting Y. pestis against Na^+^ toxicity in blood.

The *y3553* gene product is predicted to belong to the PYR/PYL/RCAR-like family of proteins, a part of the SRPBCC (START/RHO_alpha_C/PITP/Bet_v1/CoxG/CalC) domain protein superfamily, that are known to bind hydrophobic ligands and mediate signal transduction (33, 34). PYR/PYL/RCAR proteins are particularly well characterized in plants, where they bind phytohormones that enable the regulation of growth, development, and environmental stress responses. For example, osmotic stress responses and high salt tolerance are mediated by PYR/PYL/RCAR protein binding interactions with plant phytohormones (34–36).

The gene product of the *y355x* gene is predicted to be a transcriptional regulator because it contains a helix-turn-helix motif and Per-ARNT-Sim (PAS) sensory domain. PAS domains are ubiquitous in bacterial proteins involved in signal transduction and enable sensing of physical and chemical stimuli, like oxygen and redox potential (37).

The *y3551* and *y3550* gene products belong to the reactive intermediate deaminase (Rid) protein superfamily, originally called the YjgF/YER057c/UK114 family of proteins. Rid enzymes eliminate reactive intermediates that are produced by PLP-dependent enzymatic activity, which can alter the function of cellular targets and prevent the inhibition of transaminases (38–40). Interestingly, some Rid-encoding genes cluster with genes encoding PLP-dependent enzymes, like aminotransferases, which are susceptible to damage by the enamine 2-aminoacrylate (2AA) (41). The clustering of *y3551* and *y3550* with *y3555*, predicted to encode an aminotransferase, is consistent with this clustering pattern.

### The genes *y3555*, *y3554*, *y3553*, *y355x*, *y3551,* and *y3550* are transcribed as a polycistronic mRNA.

Gene clustering in bacteria often implies a functional association of its member genes. Previous transcriptomic analyses have shown that the *y3555*, *y3554*, *y3553*, *y3551,* and *y3550* genes exhibit coordinated gene expression in the flea gut and under similar *in vitro* conditions (12, 17, 24). To determine if the coordinated expression of this genomic island can occur from a single long polycistronic mRNA, a two-step reverse transcription-PCR (RT-PCR) using Y. pestis parental cDNA to amplify the entire locus from *y3555* to *y3550* (Fig. 1B) was performed. A negative control to verify the absence of genomic DNA contamination was included by omitting reverse transcriptase during cDNA synthesis (Fig. 1B, lane 7), and a positive control using Y. pestis chromosomal DNA as the template with DNA polymerase verified the expected amplicon size (Fig. 1B, lane 6). As shown in Fig. 1B, a single 5,014-bp predicted fragment spanning all six genes from *y3555* to *y3550* was amplified. Together, these results confirmed that the *y3555, y3554, y3553*, *y355x*, *y3551,* and *y3550* genes are transcribed as a long single polycistronic mRNA transcript.

Next, to determine if the predicted promoters upstream of *y3555*, *y355x,* and *y3550* drive the expression of their respective downstream genes, transcriptional fusions spanning different regions of the genomic island to the *luxCDABE* reporter genes (Fig. 1C) were used. Luminescence directed by the plasmid carrying these transcriptional fusions was determined in the parental Y. pestis strain grown at 26°C to simulate conditions within the flea. Strains harboring fusions to the promoter of *y3555* (pCS::P55) and *y355x* (pCS::P5x) showed ∼10^2^- to 10^3^-fold higher levels of luminescence expression than those of the strains carrying the empty vector (pCS26-Pac) or transcriptional fusions with potential regulatory regions of *y3554* (pCS::P54), *y3553* (pCS::P53), and *y3550* (pCS::P50). This suggests that only the upstream regulatory regions of *y3555* and *y355x*, and not *y3550,* are able to promote gene transcription using flea-matched temperature testing conditions. To confirm this observation, we generated two other transcriptional fusion constructs that allowed testing of transcription directed from the predicted upstream regulatory regions of *y3555* or *y355x*. The transcriptional fusion construct pCS::P55-54 contains the region covered in pCS::P54 extended up to the regulatory region of *y3555*, and the construct pCS::P5x-51-50 contains the region of fusion pCS::P50 extended up to the regulatory region of *y355x* (Fig. 1C). The expression of the pCS::P55-54 and pCS::P5x-51-50 fusions was drastically increased and similar to that shown in fusions P55 and P5x, respectively. (Fig. 1C). This suggested that the promoter located upstream of *y3555* can drive the expression of *y3555-y3554-y3553*-*y355x-y3551-y3550* as a single operon and that *y355x-y3551-y3550* can be transcribed as another operon from the promoter located upstream of the *y355x* gene.

### Neither single-gene nor whole-genomic-island mutant strains exhibit fitness attenuation in competitive coinfection with the parental strain.

**FIG 2:**
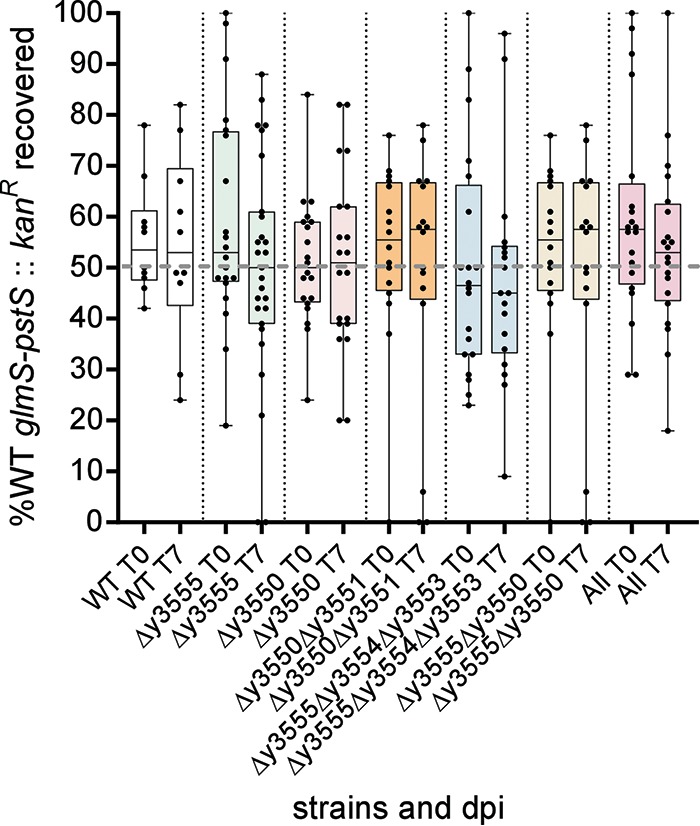
Y. pestis low-GC-content gene mutants coinfected with the parental strain do not exhibit competitive fitness defects in fleas. Fleas were infected using a 1:1 ratio of mutant to parental strains harboring a kanamycin resistance cassette in the chromosome (WT *glmS-pstS*::Kan^r^). The percent WT *glmS-pstS*::Kan^r^ from the total coinfecting Y. pestis population per flea (dots) at days 0 and 7 is reflected. The error bars represent the mean plus the minimum and maximum percent WT *glmS-pstS*::Kan^r^ per flea. The data were analyzed using an unpaired Student *t* test. dpi, days postinfection.

To test if the most highly expressed genes, *y3555* and *y3550,* contributed to efficient colonization and formation of proventricular blockage of the flea, we constructed and tested mutants lacking the *y3555* or *y3550* gene alone, *y3551* and *y3550*, *y3550* and *y3555*, and the entire locus comprising *y3555-y3554-y3553-y355x-y3551-y3550* (referred to as the “all” mutant). A previously reported *y3555-y3554-y3553* mutant was also used (42). The six mutants displayed parental abilities to infect and block fleas (data not shown). Observing no difference in single flea infection, a more sensitive competitive coinfection assay (43) designed to discern the fitness of a mutant strain relative to the parental strain was performed. Fleas were coinfected with a 1:1 ratio of mutant to parental strain, harboring a kanamycin resistance cassette in the nondeleterious *glmS-pstS* site and called wild type (WT) *glmS-pstS*::Kan^r^, which was previously shown to exhibit equal fitness during competition with an unmarked parental strain (43). All mutants tested in coinfections with the *glmS-pstS*::Kan^r^ WT strain were maintained at rates equal to that of the *glmS-pstS*::Kan^r^ WT, as reflected by constant occurrence of the *glmS-pstS*::Kan^r^ strain population at a mean of ∼50% of the total population of coinfecting Y. pestis immediately after infection (T0) and at 7 days (T7) postinfection (Fig. 2). These *in vivo* assays showed the deletion mutations did not have attenuated fitness in the flea.

### Predicted promoters located upstream of *y3555*, *y355x,* and *y3550* are responsive to hyperosmotic salinity stress.

**FIG 3:**
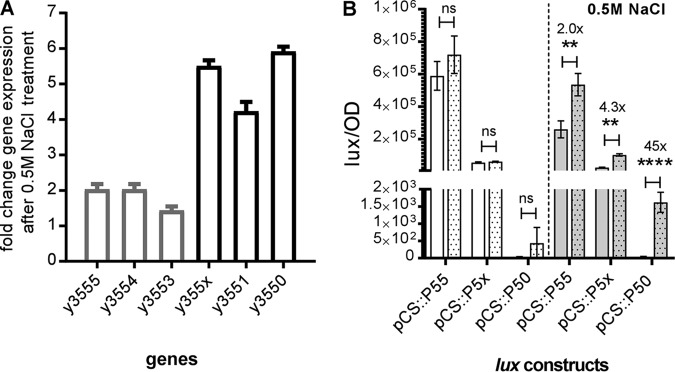
Gene expression of low-GC-content genes is induced during exposure to saline hyperosmotic stress. (A) Steady-state mRNA transcript levels of low-GC-content genes in a Y. pestis KIM6^+^ parental strain treated or not treated with 0.5 M NaCl were determined. The data are represented as the fold change in gene expression of treated versus untreated mRNA transcript levels. Error bars represent the mean (±SD) of the results from 3 independent experiments. (B) Activities of the transcriptional fusion plasmids pCS::P55, pCS::P5x, and pCS::P50 carrying the transcriptional fusions of the luciferase genes and predicted promoter regions of *y3555*, *y355x*, *y3550*, respectively, pre-treatment with vehicle (white bars) or 0.5 M NaCl (gray bars) and 100 min post-treatment with vehicle (white dotted bars) or 0.5 M NaCl (gray dotted bars). Error bars represent the mean (±SD) of the results from three independent experiments. Data were analyzed using Student's *t* test; **, *P* < 0.01; ****, *P* < 0.0001; ns, not significant.

In a previous study, the genome-wide transcriptional responses of the enzootic strain Y. pestis bv. Microtus to hyperosmotic stress mediated by high salt found that genes homologous to the Y. pestis KIM6^+^ *y3555*, *y3554*, *y3553*, *y355x*, *y3551,* and *y3550* genes are upregulated (24). This study suggested that the genomic island is responsive to hyperosmotic stress. To determine if the *y3555, y3554, y3553, y355x, y3551*, and *y3550* genes were induced in response to hyperosmolarity stress in the KIM6^+^ epidemic strain, we exposed this strain to 0.5 M NaCl (reflects a high osmolarity of 1,000 mOsm) and determined the steady-state mRNA expression levels of these genes. The expression levels for the genes *y3555, y3554,* and *y3553* showed between 1.5- and 2.0-fold increases, while the expression of the genes determined to compose the second operon, i.e., *y355x-y3551-y3550*, showed between 4- and 6-fold increases in gene expression (Fig. 3A).

Additionally, the induction of *lux* transcriptional reporter fusion activity was assessed for the pCS::P55, pCS::P5x, and pCS::P50 transcriptional fusion reporter constructs to determine if reporter activity increased after NaCl exposure. This time readings were taken 100 min after exposure to 0.5 M NaCl (Fig. 3B). Interestingly, while the Lux reporter activity from pCS::P55 and pCS::P5x showed 2- and 4-fold increases, respectively, a remarkable 45-fold increase in Lux reporter activity from pCS::P50 was observed following exposure to 0.5 M NaCl. These data indicated that, while the promoters upstream of *y3555* and *y355x* promote transcription at flea-matched temperatures, they exhibit increased transcriptional activity under hyperosmotic/saline conditions, whereas the promoter upstream of *y3550* is active only under hyperosmotic/saline conditions. RT-PCR analysis (Fig. 1B) indicates that the genomic island continues to be expressed as a single long transcript following exposure to 0.5 M NaCl.

### Y3555 generates a critical requirement for aspartate in a Salmonella enterica *aspC* mutant.

The *ridA* and *aspC* genes, encoding aspartate aminotransferases, cluster in many bacterial chromosomes (41). Some online genome resources, including KEGG, Phyre2 (44), and BLAST, predict that Y3555 is an aspartate aminotransferase, and for this reason, aspartate was considered as a possible substrate for the enzyme. However, based on structural alignments, it appears that Y3555 has a glutamate instead of an aspartate residue in the active site to coordinate the pyridine nitrogen of PLP. These initial alignments suggest Y3555 may be a fold type IV aminotransferase (45), not a fold type I aminotransferase like AspC (45) (see Fig. S1 in the supplemental material).

**FIG 4:**
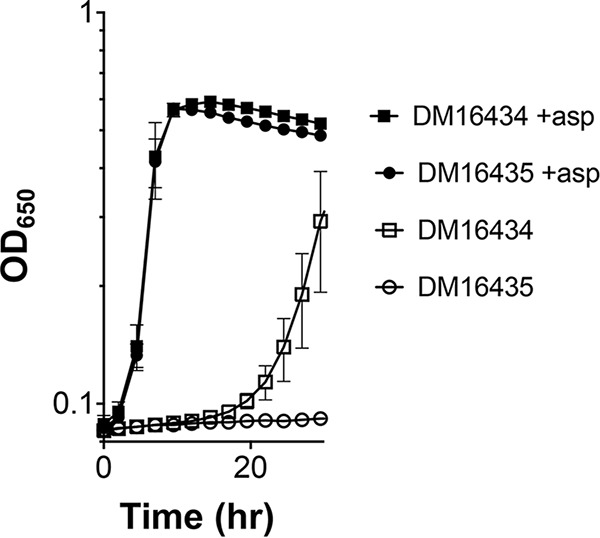
Induction of *y3555* generates an aspartate requirement for an S. enterica *aspC789*::Km mutant. S. enterica *aspC789*::Km mutants expressing *y3555* DM16435 or the empty vector control DM16434 were grown in minimal glucose (11 mM) medium plus IPTG (100 μM), with (+asp) or without aspartate (1 mM). Error bars represent the mean (±standard error of the mean [SEM]) values from three biological replicates.

Nonetheless, to experimentally query if Y3555 had indeed predicted aspartate aminotransferase activity, the ability of *y3555* to restore the growth of an S. enterica *aspC789*::Km mutant strain was tested. Growth of the S. enterica *aspC789*::Km mutant containing either the empty pTRc99A plasmid or plasmid pTRc_55, carrying an isopropyl-β-d-1-thiogalactopyranoside (IPTG)-inducible *y3555* gene, was determined (Fig. 4). The S. enterica *aspC789*::Km mutant has a growth defect in glucose minimal medium, since it relies on low-level promiscuous aspartate aminotransferase activity of the tyrosine aminotransferase TyrB. The S. enterica *aspC789*::Km mutant strains containing either the empty pTRc99A plasmid or plasmid pTRc_55 had similar growth on minimal glucose medium with aspartate. Expression of the *aspC* gene from S. enterica allows full growth of the mutant strain (Fig. S2). Surprisingly, in the absence of aspartate, the addition of 100 μM IPTG, presumed to induce the expression of *y3555*, inhibited the growth of the S. enterica *aspC789*::Km mutant (Fig. 4). This growth defect was eliminated with the addition of aspartate, suggesting that the critical aspartate requirement was facilitated by Y3555 expression. There are a few possibilities for this effect that cannot be distinguished by the data here, including that Y3555 could affect TyrB flux or produce tyrosine and phenylalanine that shut down TyrB expression and/or activity (46–48).

### Y3551, but not Y3550, is a functional RidA deaminase.

**FIG 5:**
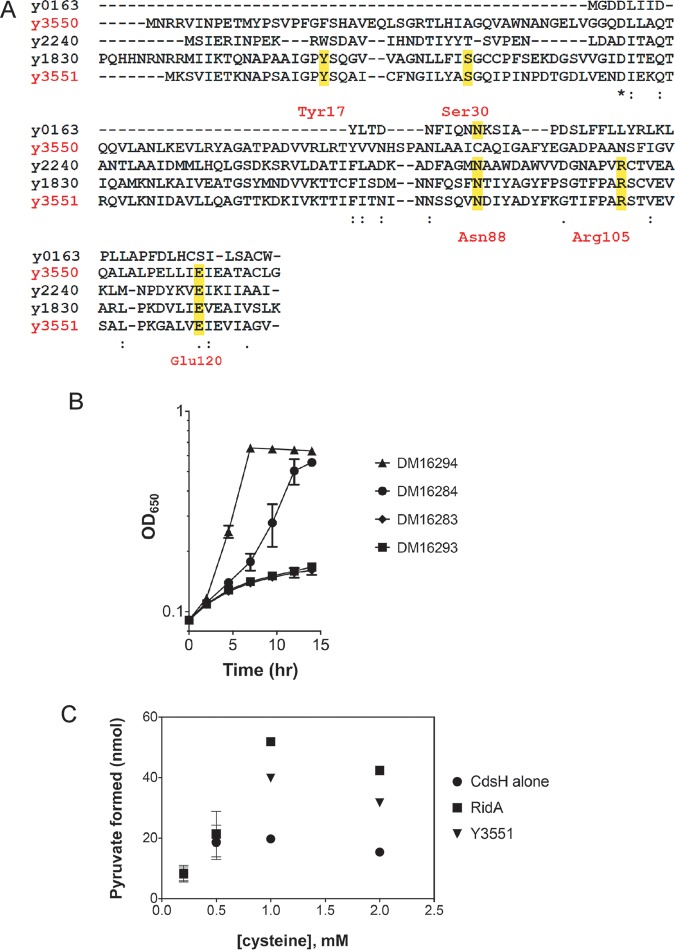
Y3551 is a RidA enzyme. (A) Multiple-sequence alignment of all Y. pestis KIM6^+^ Rid family homologs. Amino acids highlighted in yellow represent conserved residues found in archetypal RidA subfamily members. *y3551* complements an S. enterica *ridA* mutant. (B) The S. enterica *ridA1*::Tn*10* (Tc) mutant carried the empty vector control DM16293 or plasmid pET28b expressing E. coli *ridA* DM16294, *y3551* DM16284, or *y3550* DM16283. Strains were grown in a 96-well plate at 37°C shaking in minimal NCE glucose (11 mM) with serine (5 mM). Error bars represent the mean (±SEM) from three biological replicates. (C) Y3551 deaminates 2-aminoacrylate *in vitro*. Purified S. enterica cysteine desulfhydrase (CdsH) was used to generate 2AA from cysteine. Reactions with or without the addition of Y3551 or S. enterica RidA measured coupled pyruvate formation and NADH oxidation, as previously described (Ernst et al. [67] and Kredich [69]). NADH oxidation was used as a measurement of pyruvate formation. RidA enzymes deaminate 2AA *in vitro*, increasing the reaction rate. The initial reaction rate of pyruvate formation is plotted versus the concentration of cysteine. As a control, CdsH alone or S. enterica RidA was used. Y3551 deaminated 2AA derived from cysteine. Error bars represent the standard error of the mean from technical triplicates.

Proteins of the Rid superfamily are defined as belonging to the archetypal RidA subfamily or Rid1 to -7 subfamilies based on phylogenetic analysis (41). RidA enzymes hydrolyze reactive imine/enamine metabolic intermediates (i.e., 2-aminoacrylate [2AA]) (40), which are generated as obligate catalytic intermediates by some PLP-dependent enzymes. An active-site arginine (Arg105) is critical for RidA imine hydrolysis activity (40). Rid4 to -7 subfamily members lack the active-site arginine and an ascribed biochemical function. Amino acid alignment of the Y3550 and Y3551 sequences with Rid family homologs (Fig. 5A) revealed that Y3551 had the requisite arginine in the active site. Y3550 is missing an active-site arginine, contains a phenylalanine at position 17 rather than the typical tyrosine, and has a variable asparagine at position 88. These features of Y3550 conform to those described previously for Rid5 or Rid6 proteins (41).

To test if Y3551 and/or Y3550 had the deaminase activity conserved for RidA enzymes, the S. enterica *ridA1*::Tn*10* (Tc) mutant strain was transformed with the plasmid constructs pET28b::*ridA*, pET28a::*y3551,* and pET28a::*y3550*. Minimal defined medium supplemented with serine was used for growth analysis. In the S. enterica *ridA1*::Tn*10* (Tc) mutant, 2AA is generated from serine by the serine/threonine dehydratase IlvA (EC 4.3.1.19). In the absence of RidA, 2AA accumulates and damages some PLP-dependent enzymes, which can result in growth defects (49). Growth was monitored to determine if Y3551 and/or Y3550 could restore growth to the S. enterica *ridA1*::Tn*10* (Tc) mutant. The S. enterica *ridA1*::Tn*10* (Tc) mutant strain carrying the empty vector control failed to grow in this medium, and as expected, it was complemented by the pET28b::*ridA* plasmid. Significantly, the pET28a::*y3551* plasmid restored significant growth to the strain, indicating that Y3551 had 2AA deaminase activity (Fig. 5B). However, the pET28a::*y3550* plasmid failed to restore growth to the S. enterica *ridA1*::Tn*10* (Tc) mutant strain (Fig. 5B). Control experiments confirmed that the addition of isoleucine restored full growth to all strains, confirming that the weak growth was due to 2AA accumulation (data not shown).

To confirm that Y3551 had 2AA hydrolysis activity, as indicated by the complementation analyses, assays were done with pure proteins. Cysteine desulfhydrase (CdsH) converts cysteine to 2AA, which can be deaminated to pyruvate by solvent water or a RidA enzyme. Thus, RidA activity is detected as an accelerated rate of pyruvate formation in a CdsH assay. The assay was performed using purified Y3551 and S. enterica RidA enzymes, and the data are shown in Fig. 5C. In the presence of either purified S. enterica RidA or Y3551, the initial rate of pyruvate formation was accelerated over the control lacking RidA when the cysteine concentration was 1 mM or greater (Fig. 5C). These data confirmed that Y3551 is a RidA family enzyme with 2AA hydrolysis activity.

One other disparate biochemical activity previously assigned to Rid enzymes is that of an endoribonuclease (41, 50). The Y3551 and Y3550 proteins were in fact previously designated l-PSP family endonucleases by BLAST analyses (12, 17). However, our purified Y3551 and Y3550 did not show any RNase activity (data not shown), supporting the idea that these proteins do not encode such a function in Y. pestis.

### Overexpression of *y3555* enhances the growth of E. coli and Y. pestis.

**FIG 6:**
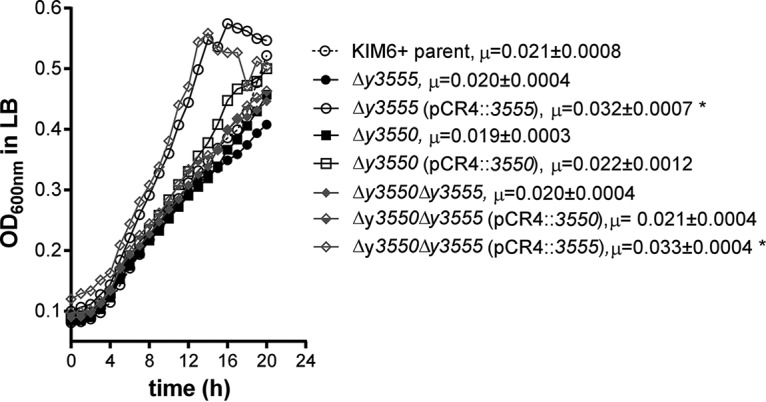
Overexpression of *y3555* increases the growth fitness of Y. pestis strains. Shown are the growth kinetics of the Y. pestis KIM6^+^ parental strain, Δ*y3550* mutant, Δ*y3555* mutant, and Δ*y3550* Δ*y3555* mutant alone or overexpressing either *y3555* (pCR4::*3555*) or *y3550* (pCR4::*3550*) in LB medium. Data represent the mean values of the results from three independent experiments. The growth rate (μ) calculated from regression analysis is given next to each strain name. Data were analyzed using one-way ANOVA and a Tukey *post hoc* test. Asterisks mark significantly different growth rates of mutant strains versus that of the parental strain.

**FIG 7:**
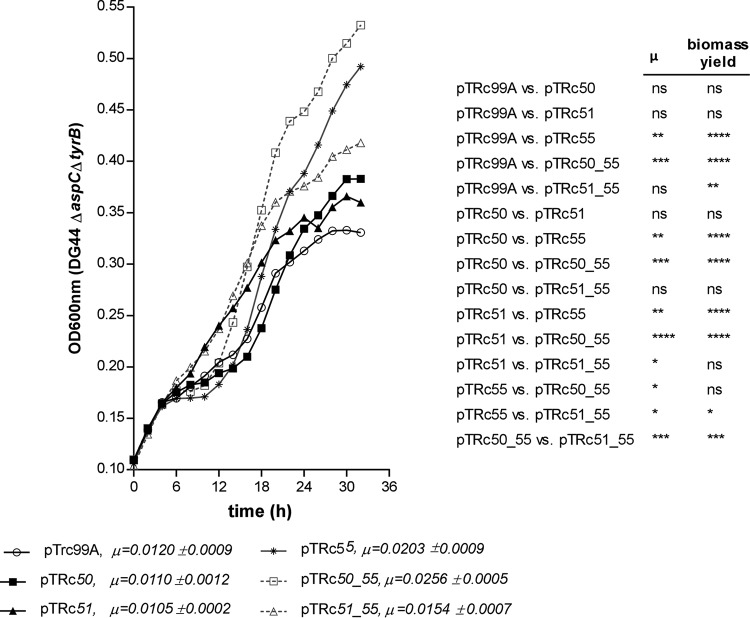
Heterologous expression of *y3555* increases the growth rate of the Escherichia coli Δ*aspC* Δ*tyrB* (DG44) mutant. Shown are the growth kinetics of E. coli DG44 in M9 minimal medium supplemented with 0.4% glucose, 0.1% Casamino Acids, succinate (2.5 mg/ml), malate (2.5 mg/ml), and α-ketoglutarate (1 mg/ml), as well as 100 μg/ml each of asparagine, glutamine, and glutamic acid. The growth rate (μ) is given next to each plasmid name. Error bars represent mean values of the results from seven independent replicates for the E. coli DG44 studies. One-way ANOVA and Tukey’s multiple-comparison test were used to determine significant differences in growth rate and final biomass yield. *, *P* < 0.05; **, *P* < 0.01; ***, *P* < 0.001; ****, *P* < 0.000; ns, not significant.

The uncharacterized protein Y3555 enhanced the growth of Y. pestis strains grown in rich medium. The Y. pestis *y3555* and *y3550* mutants expressing Y3555 from a high-copy-number plasmid (pCR4-TOPO) grew significantly faster than did the wild-type strain grown under the same conditions (Fig. 6). A similar observation was made in an E. coli aspartate (*aspC*) and tyrosine (*tyrB*) aromatic aminotransferase double mutant that requires either aspartate or tyrosine for growth (46, 51). This strain, called DG44, was used in early trial experiments replicating those in the S. enterica *aspC359*::Km mutant, but those experiments were abandoned due to a lack of genetic complementation tools. It has been suggested that Rid enzymes may be encoded by genes for PLP-dependent enzymes that are targets of 2AA damage, including branched-chain amino acid transaminases and aspartate aminotransferases (41). These reports, along with the gene synteny of *y3550*, *y3551*, and *y3555,* suggest that either *y3551* or *y3550* may prevent the accumulation of a toxic metabolic intermediate of *y3555*. The improvement in growth observed with the overexpression of *y3555* presented an opportunity to test if *y3550* or *y3551* expression combined with that of *y3555* could further improve the growth kinetics of the DG44 strain. The DG44 strain was transformed with inducible plasmids carrying a single *y3555*, *y3550,* or *y3551* gene and either the *y3555* and *y3550* genes or the *y3555* and *y3551* genes. Significantly higher growth rate and biomass yield were observed for the DG44 strain expressing *y3555* (μ = 0.0203 ± 0.0009) but not *y3550* (μ = 0.0110 ± 0.0012) and *y3551* (μ = 0.0105 ± 0.0002) relative to the empty vector-containing strain (μ = 0.0120 ± 0.0009) (Fig. 7). The coexpression of *y3555* with *y3550* (μ = 0.0256 ± 0.0005) or *y3551* (μ = 0.0154 ± 0.0007) increased the growth rate and biomass yield similar to those of the *y3555*-expressing strain, indicating that the increased growth rate and yield were *y3555* dependent (Fig. 7). However, the highest growth rates occurred in the strain coexpressing *y3550* and *y3555,* followed by the strain expressing *y3555* alone, and next, the strain coexpressing *y3555* and *y3551*. The *y3550*- and *y3555*-coexpressing strain achieved overall higher biomass yield than did all other strains tested except the *y3555*-expressing strain. Strains expressing *y3550* or *y3551* alone had growth rates and biomass yields similar to those of the empty vector-containing strain.

## DISCUSSION

In this work, we have determined that the coordinated expression of an uncharacterized cluster of low-GC-content genes, *y3555-y3554-y3553-y355x-y3551-y3550*, that show exceptionally robust expression during flea infection, is accounted for by their cotranscription as a single polycistronic mRNA transcript. Adjacently clustered genes are often grouped transcriptionally as an operon and frequently encode functionally related proteins. This feature allows protein synthesis to be controlled coordinately in response to the needs of the bacterium only when and where the proteins are required. While the genomic island is expressed only at flea-matched temperatures (16, 17), the further induction of these genes following exposure to hyperosmotic saline stress implicates these genes as being functionally concerned with osmoadaptation during the Y. pestis flea life stage. Indeed, protein homology for the *y3553* and *y3554* gene products clearly predicts that these proteins have direct roles in hypersaline osmotic stress adaptation. In the case of Y3553, active exchange of a sodium ion for a proton is predicted, whereas Y3554 is likely involved in transducing osmotic stress signals to mediate osmoadaptation. However, disruption of the genomic island in the “all” mutant did not result in any survival defects after exposure to 0.5 M NaCl during *in vitro* LB broth culture, as this mutant survived at levels comparable to those of the parental Y. pestis strain (data not shown). This suggested that absence of the genomic island is not critical for adaptation to osmotic stress *in vitro*. Nonetheless, *y3555,* a metabolic gene showing putative roles in amino acid metabolism, confers a striking ability to enhance the growth fitness of E. coli, a trait that is also made apparent during the overexpression of *y3555* in Y. pestis strains. These two findings allude to a role of the genomic island in optimizing amino acid metabolism in the context of hyperosmotic stress. This is fitting since (i) Y. pestis primarily metabolizes amino acids during flea infection (15–17) and (ii) the flea gut has a demonstrated physicochemical status of high osmolarity (11), while several Y. pestis metabolic pathways involved in osmoprotection are transcriptionally induced during flea gut infection (15, 17).

To begin to understand the function of Y3555, it was expressed in *trans* in a well-characterized S. enterica *aspC* mutant. An S. enterica *aspC* mutant has a growth defect that is restored with the addition of aspartate or *aspC* expressed in *trans*. However, when Y3555 was expressed in the S. enterica *aspC359*::Km mutant, growth was not restored but in fact further retarded. This surprising result may, however, hint at the function of Y3555. There is evidence to show that in an *aspC* mutant of S. enterica or E. coli, TyrB produces minimal aspartate that allows for some growth (48, 52), but the addition of tyrosine or phenylalanine inhibits *tyrB* expression and growth (51). As such, one explanation for the growth defect of the S. enterica *aspC359*::Km mutant expressing Y3555 is that Y3555 increases tyrosine and/or phenylalanine pools that lead to *tyrB* inhibition. Thus, our findings suggest that while Y3555 is not an aspartate aminotransferase, it could instead be an aromatic aminotransferase. Alignments of Y3555 with AspC and TyrB from both S. enterica and Y. pestis suggest that Y3555 proteins are fairly dissimilar (see Fig. S1 in the supplemental material). Instead, Y3555 aligns with over 85% identity to uncharacterized PLP-dependent aminotransferases from foodborne isolates of S. enterica sequenced by the Centers for Disease Control and Prevention (Fig. S3). The crystallization of Y3555 may help determine the substrates or products of the protein, and their characterization is ongoing.

During active metabolism, cellular function can be compromised by an anomalous accumulation of endogenously produced reactive intermediates of metabolism, e.g., 2AA. The elimination of such metabolic intermediates is therefore needed to optimize cellular function, growth, and fitness. The highly conserved Rid family proteins prevalent in all domains of life (41, 49) are among the first enzymes characterized to support the optimization of metabolic processes by neutralizing obligatory reactive metabolic intermediates. Currently, RidA enzymes are well characterized as detoxifiers of 2AA in several bacterial species (38, 40, 41, 49, 53). Our data contribute to this list by establishing that in Y. pestis, the *y3551* gene encodes an archetypal RidA protein that can deaminate the reactive enamine intermediate 2AA. Additionally, the Y3550 gene product likely belongs to the Rid5-6 subclass of the Rid family proteins whose functions have not yet been characterized. Further adding to its distinctive status is that while the promoters predicted immediately upstream of *y3555* and *y355x* can drive the transcription of *y3550*, a dedicated promoter predicted immediately upstream of the Y3550 coding sequence is exclusively activated by hyperosmotic stress to inclusively drive *y3550* transcription.

It remains elusive how Y3555, Y3550, and Y3551 operate collaboratively to achieve optimal metabolic activity in response to osmotic stress. The achievement of increased growth rate in the DG44 strain coexpressing *y3555* and *y3550* suggests a synergistic activity of these two proteins. Such a case can be explained by the specific Rid activity meted out by Y3550 being able to potentially eliminate a metabolic by-product that is toxic and/or produced by Y3555. One complementary observation within a context of Y3555 serving in aromatic amino acid metabolic processes is that BPROM predicts a binding box of the transcriptional factor TyrR (54–56) in the promoter sequence of *y3550* (the predicted promoter region spans nucleotide positions −120 to −87). In many enteric bacteria, TyrR regulates genes involved in the uptake and biosynthesis of aromatic amino acids in response to the presence or absence of each of the three aromatic amino acids (tyrosine, tryptophan, and phenylalanine) which function as cofactors for TyrR (54, 55). For example, in E. coli, TyrR is responsive to tyrosine and able to regulate the expression of tyrosine biosynthesis genes *tyrB*, *aroF-tyrA,* and *aroLM* and genes encoding aromatic amino acid transporters, *aroP* and *mtrR* (55).

Conversely, the suppressed growth rate in the DG44 strain coexpressing *y3555* and *y3551* versus that of *y3555* expression alone suggests antagonistic activity of these two encoded proteins. Such a scenario can be explained by Y3551 RidA deaminase activity protecting an unknown enzyme whose activity either competes with or generates reactive intermediates to which Y3555 is susceptible. The coexpression of Y3551 RidA and Y3550 in Y. pestis might therefore accomplish a balance in metabolic fitness as it pertains to aromatic amino acid metabolic activities.

Our experimental data demonstrate that mutations in the genomic island afford no compromise in the growth and survival or competitive fitness of Y. pestis in fleas. This is confounding because highly elevated expression of unrequired genes would be energetically expensive if not required in the flea gut environment. It is possible that controlled lab-reared flea experiments cannot discern a crucial function of gene products that in wild fleas feeding on wild rodent blood sources (different from lab mouse species) is otherwise apparent. It has been demonstrated that the infectious blood source influences Y. pestis infection in fleas (57) and that the gut microbiota of wild fleas is more diverse than that of lab-reared fleas (58). Perhaps in the wild, a competitive coinfection arises between Y. pestis and diverse gut microbes necessitating genomic island gene product function to heighten its competitive fitness.

Moreover, it is known that more than a single pathway contributes to metabolic robustness, and metabolic versatility is tolerated in the repertoire of metabolic gene pathways that might be switched on for a common physiological purpose, e.g., sustaining Y. pestis growth and survival in the flea gut environment under hyperosmotic stress conditions. For example, the osmoprotectant glycine-betaine is produced by *proVW* and *betI,* and these genes have been shown to be expressed by Y. pestis during flea infection (15). Additionally, the Y. pestis genome encodes homologs of some members of the genomic island. Two PLP-dependent aromatic aminotransferase genes, *y2760* (*aspC*) and *y0579* (*tyrB*), which convert aspartate and tyrosine to glutamate, respectively, are induced at robust levels during a Y. pestis flea infection (17). The *y3554* gene is one of three genes encoding a Na^+^/H^+^ antiporter (*nhaA*, *nhaB*, and *nhaC*). Two *ridA* homologs, *y2240* and *y1830*, and a putative Rid1 to -7 superfamily-encoding gene, *y0163,* may be able to compensate for the functions of their homologs in the genomic island (Fig. 5A).

Overall, our data reinforce a model wherein once Y. pestis enters the flea in the blood meal, it transitions into an environment of elevated osmotic stress (11) likely exacerbated by water removal from the blood meal during flea digestive processes (59). Osmotic pressure in the blood meal is contributed by the salts it contains, the concentrations of which are expected to increase after water removal. The requirement for NhaB and NhaC Na^+^/H^+^ antiporters for the virulence of Y. pestis during septicemic infection supports the idea that this bacterium faces hyperosmotic stress in mammalian blood (31, 32). Simultaneously, Y. pestis must optimize its growth fitness to establish a transmissible infection in the hyperosmotic flea gut environment. The mechanisms by which Y. pestis counterbalances osmotic stress in the flea, however, remain unclear, although our current data suggest that this is linked to amino acid metabolism shifts that support osmoadaptation within the flea gut. Interestingly, Y. pestis encodes and expresses several biochemical and regulatory pathways that function in alleviating osmotic stress, emphasizing (11, 15) that osmotic stress is a perilous stress to which Y. pestis must adapt in the flea gut. Preservation of compensatory/redundant osmotic stress resistance pathways that allow simultaneous optimization of Y. pestis growth fitness to establish a transmissible infection in fleas as such is critical for the maintenance of disease cycles.

## MATERIALS AND METHODS

### Bacterial strains and growth conditions.

The bacterial strains used in this work are listed in Table S1 in the supplemental material. Bacterial cultures were grown at 28°C with shaking in brain heart infusion (BHI) medium. When necessary, media were supplemented with carbenicillin (100 μg/ml) or kanamycin (50 μg/ml). For the osmolarity assays, overnight cultures of parental KIM6^+^ strains were used to inoculate fresh LB medium and were incubated at 28°C to reach mid-logarithmic phase; thereafter, cultures were exposed to 0.5 M NaCl. Bacteria were immediately mixed with RNAprotect bacterial reagent (Qiagen) to minimize RNA degradation and then harvested at 4°C and stored at −80°C until RNA isolation.

Salmonella enterica serovar Typhimurium LT2 was the parental strain for all S. enterica mutants. Overnight cultures of S. enterica were grown in 8 g/liter Difco nutrient broth (NB) with NaCl (5 g/liter) and the appropriate antibiotic (150 μg/ml ampicillin or 50 μg/ml kanamycin). Minimal no-carbon E (NCE) was supplemented with MgSO_4_ (1 mM), trace minerals, glucose (11 mM), and, where indicated, serine (5 mM), isoleucine (0.3 mM), aspartate (1 mM), and/or isopropyl-β-d-1-thiogalactopyranoside (IPTG; 100 μM) (60, 61). All chemicals were purchased from the Sigma-Aldrich Chemical Company in St. Louis, MO.

### Construction of plasmids.

The plasmids and primers used in this study are listed in Tables S1 and S2, respectively.

### Lux transcriptional fusion constructs.

The Lux transcriptional fusions were generated by cloning a PCR product containing the corresponding regulatory region into the low-copy-number vector pCS26-Pac that carries the promoterless *luxCDABE* operon (62). PCR products were obtained with the 50R-BHI/50F-XhoI, 51R-BHI/51F-XhoI, 50R-BHI/51F-XhoI, 53R-BHI/53F-XhoI, 54R-BHI/54F-XhoI, 55R-BHI/55F-XhoI, and 54R-BHI/55F-XhoI primer pairs (Table S2). They were then digested with BamHI and XhoI restriction enzymes (NEB) and cloned into the pCS26-*Pac* plasmid previously digested with the same restriction enzymes, generating plasmids pCS::P50, pCS::P5x, pCS::P5x_51_50. pCS::P53, pCS::P54, pCS::P55, and pCS::P55_54, respectively (Table S1). Lux transcriptional fusion plasmids were transformed into the Y. pestis KIM6^+^ parental strain.

### pTRc99A and pET28a constructs.

The pTRc99A- and pET28a-inducible expression plasmids were generated by cloning PCR products obtained for the *y3550*, *y3551,* and *y3555* genes from the p270/p271, p272/p273, and p274/p275 primer pairs, respectively. The PCR products were digested with BamHI and XhoI and ligated into pTrc99A or pET28a previously digested with BamHI and SalI restriction enzymes. The plasmid constructs generated from cloning *y3550*, *y3551,* and *y3555* into pTRc99A are referred to as pTRc55, pTRc50, and pTRc51, and for pET28a cloning, they are referred to as pET28a::*y3550*, pET28a::*y3551,* and pET28a::*y3555,* respectively. To clone *y3555* together with *y3551* or *y3550* into pTrc99A, *y3555* was amplified with primers p508 and p509, digested with HindIII, and ligated into pTRc50 or pTRc51 such that *y3555* was inserted downstream of *y3550* or *y3551*. These plasmids were referred to as pTRc50_55 and pTRc51_55, respectively. Plasmids were transformed into transformed into E. coli DG44 and selected for on LB–100 μg/ml carbenicillin.

### Tn*7*-based chromosomal integration of a kanamycin resistance cassette into Y. pestis KIM6^+^ strain.

The kanamycin resistance cassette from the plasmid pKD4 was amplified with primers p395 and p396 (Table S2). The PCR product was digested with BamHI and EcoRI, ligated into pUC18R6K-mini-Tn*7*T previously digested with the same restriction enzymes, transformed into DH5α lambda *pir* chemical competent E. coli, and selected for on LB–50 μg/ml kanamycin. The plasmid, referred to as pUC18R6K-mini-Tn*7*T::*kan*^r^, was isolated and verified for sequence. Electrocompetent Y. pestis KIM6^+^ was transformed with pUC18R6K-mini-Tn*7*T::*kan*^r^ and pTNS2 and selected for on heart infusion broth (HIB)–50 μg/ml kanamycin plates. Colonies were screened with the pstSup2 and pTn7R primers for insertion at the *glmS-pstS* site. This Y. pestis KIM6^+^ strain with the kanamycin cassette integrated at the *glmS-pstS* site was referred to as Y. pestis KIM6^+^ *glm-pstS*::Kan^r^.

### Lux transcriptional reporter assay.

Y. pestis KIM6^+^ carrying plasmids containing the *lux* fusions was grown overnight in LB supplemented with appropriate antibiotics. An aliquot was used to inoculate fresh LB medium the next day. The cultures were grown on a shaker at 28°C to mid-logarithmic phase and then treated or not with 0.5 M NaCl for 100 min. Aliquots of 1 ml were taken out to determine the optical density of the culture by measuring the optical density at 600 nm (OD_600_). One-tenth of a milliliter of each culture was dispensed into a 96-well plate (Costar), and the luminescence of each well was measured in the Infinite M1000 reader (Tecan), with an integration time of 1 s and using the Tecan i-control software version 1.7.1.12. Plates were first shaken with a 2-mm linear amplitude 5-s pulse. Data were normalized by dividing relative light units (RLU) by the OD_600_ to obtain the final RLU corrected for bacterial growth.

### RT-PCR.

The Y. pestis KIM6^+^ strain was grown in Miller’s LB broth at 26°C to mid-logarithmic phase; the culture was split in two, and one-half received 0.5 M NaCl and the other distilled water vehicle control treatment. After 20 min, culture RNA was stabilized immediately with RNAprotect bacterial reagent (Qiagen). Total RNA was purified using the Quick-RNA miniprep kit (Zymo). Purified RNA was subject to Turbo DNase I (Ambion) treatment, according to the manufacturer’s instructions. Then, 800 to 1,600 ng of RNA was used in the first-strand cDNA synthesis reaction using Maxima H Minus reverse transcriptase (Thermo Fisher), according to the manufacturer’s instructions. Negative controls were performed by omitting the reverse transcriptase. The cDNA was then treated with RNase H (NEB) at 37°C for 30 min. The PCR amplification step was performed using Phusion polymerase (Thermo Fisher), 100 ng of cDNA, and the Y3555Set2Fw/5051RT-R primer pair (each primer at a final concentration of 0.5 μM) (Table S2); this pair amplifies a 5,014-bp region upstream of *y3555* to 226 bp within *y3550*. RT-PCR cycles were as follows: one cycle of 1 min at 98°C for initial denaturation, and 31 cycles of 10 s at 98°C, 20 s at 68°C, and 1 min 40 s at 72°C for PCR amplification with the Y3555Set2Fw/5051RT-R primer pair. A final elongation step of 5 min at 72°C was included. Controls for the expected size of the PCR products were performed by using chromosomal DNA from Y. pestis KIM6^+^ as the template. The PCR products were analyzed by 1% agarose gel electrophoresis gels. The GeneRuler 1-kb DNA ladder (Thermo Scientific) was run alongside the PCR products.

### Construction of mutant and complementation strains.

Nonpolar gene deletion mutant strains were generated by the lambda (λ) red recombinase system (63, 64) and gene-specific primer pairs shown in Table S2. The *y3550* and *y3555* genes and the entire continuous region spanning genes *y3555*, *y3554*, *y3553*, *y355x*, *y3551,* and *y3550* (referred to as “all”) and the tandem genes *y3551* and *y3550* were replaced with a selectable kanamycin (Km) resistance cassette in the Y. pestis KIM6^+^ strain (Table S1). The Δ*y3550* Δ*y3555* double mutant was constructed similarly, but for this, the Δ*y3550* mutant was used. Then, the FLP recombination target (FRT)-flanked Km cassette was excised from these strains after transformation with the helper plasmid pFLP3 (Table S1), expressing the FLP recombinase that acts on the directly repeated FRT (65). Both pKOBEG-*sacB* and pFLP3 were cured by sucrose counterselection by growing the strains on LB without NaCl and supplemented with 10% sucrose. The strains generated were Δ*y3550* (VV150), Δ*y3555* (VV151), Δ*y3550* Δ*y3551* (VV278), Δ*y3550* Δ*y3555* (VV279), and “all” (VV280) mutants (Table S1) and retained an FRT scar. To construct plasmids expressing *y3550* or y*3555,* a DNA fragment containing the *y3550* gene alone or *y3555* with its native promoter sequence was amplified by PCR using the 3550-F/3555-R or 3550-F/3555-R primer pair (Table S2) and cloned into vector pCR4-TOPO, generating plasmids pCR4::3550 and pCR4::3555, respectively. All mutant strains were verified by PCR and sequencing.

### Reverse transcriptase qPCR.

Total RNA of Y. pestis KIM6^+^ was purified using the Quick-RNA miniprep kit (Zymo). Chromosomal DNA was removed by incubating 5 μg of RNA from each strain with Turbo DNase I (Ambion), according to the manufacturer’s instructions. cDNA was synthesized in a reaction mixture containing 2 μg of each DNase-treated RNA, 100 pmol random hexamers, and 0.5 mM dinucleoside triphosphates (dNTPs), using RiboLock RNase inhibitor (Thermo Fisher) and Maxima H minus reverse transcriptase (Thermo Fisher), according to the manufacturer’s protocol. The obtained cDNA was used as the template for quantitative PCR (qPCR) assays, with 0.5 μM primer pairs for *y3550*, *y3551*, *y3553*, *y3554*, *y3555,* or *gyrB* (Table S2) and the SsoAdvanced Universal SYBR green Supermix (Bio-Rad). Real-time PCRs were performed with the c1000 Touch thermal cycler and the CFX96 real-time system (Bio-Rad), and data were collected using the CFX Manager software 2.1 (Bio-Rad). The reaction conditions were 95°C for 3 min, 95°C for 10 s, and 58°C for 30 s during 40 cycles. The level of *gyrB* mRNA was used as an internal control to normalize the results. The quantification technique used to analyze data from cultures was the 2^–ΔΔ^*^CT^* method (66). All qPCRs were performed in triplicate and were repeated using RNA purified from three independent bacterial cultures or flea infections.

### Flea infections.

For coinfections, Xenopsylla cheopis fleas were infected with blood containing a 1:1 mix of parental KIM6^+^ marked at the *attn7* site (65), called KIM6^+^ *glmS-pstS*::Kan^r^, and a mutant strain, as previously described (43). The infected fleas were fed twice weekly on a live neonatal mouse. To compare the bacterial load over the infection, 20 fleas were individually triturated and plated on BHI agar and BHI agar containing kanamycin to select for the KIM6^+^ *glmS-pstS*::Kan^r^ at 2 h (time zero) and 7 days postinfection. All experiments involving animals were approved by the Institutional Animal Care and Use Committee (IACUC) at Washington State University, Pullman, WA, and were conducted in strict accordance with institutional guidelines based on the U.S. National Institutes of Health (NIH) *Guide for the Care and Use of Laboratory Animals*.

### Growth fitness of E. coli strains DH5α and DG44 expressing Y. pestis low-GC-content genes.

E. coli strains harboring IPTG-inducible constructs were grown overnight in LB containing 100 μg/ml carbenicillin with shaking at 37°C. For the E. coli DH5α strains, the overnight inoculum was diluted 1:200 into fresh M9 medium supplemented with 0.4% glucose and 0.1% Casamino Acids medium (Difco). For the E. coli D44 strains, the overnight inoculum was diluted 1:200 into fresh M9 medium supplemented with 0.4% glucose and 0.1% Casamino Acids medium (Difco), succinate (2.5 mg/ml), malate (2.5 mg/ml), α-ketoglutarate (1 mg/ml), and 100 μg/ml each asparagine, glutamine, and glutamic acid, as previously described (46). Growth curves were generated at 37°C using a Bioscreen C shaking incubator (Growth Curves USA, Piscataway, NJ).

### Growth quantification of S. enterica strains.

The growth of S. enterica in liquid culture was assessed using a BioTek EL808 microtiter plate reader by measuring the optical density at 650 nm at 37°C at a low shaking speed. Overnight cultures of S. enterica in biological triplicate were grown in rich medium with the appropriate antibiotic at 37°C. Cultures were pelleted and resuspended in an equal volume of sterile NaCl (8.5 g/liter). The cell suspension was used to inoculate growth medium (2% inoculum), and growth was monitored. The growth data were plotted in a log_10_ format using GraphPad Prism 7.0c, and the error bars represent the standard error of the mean for three biological replicates.

### Protein purification.

Proteins were purified as previously described (53, 67, 68). _Ec_RidA and Y3551 were purified from E. coli strain BL21-AI harboring either pET20-*ridA* (DM12740) or pET28b-*y3551* (DM16279). Overnight cultures grown in superbroth with ampicillin were used to inoculate 3 liters of superbroth with ampicillin. Cultures were grown for ∼3 h at 37°C with shaking until an OD_650_ of 0.7 was reached. Fresh arabinose was added to a final concentration of 0.2%, and cultures were incubated with shaking at 37°C overnight. A clarified supernatant was injected onto a nickel-nitrilotriacetic acid (Ni-NTA) Superflow resin, and proteins were purified according to the manufacturer’s protocol (Qiagen). Protein aliquots were frozen in liquid nitrogen and stored at −80°C. Purified CdsH was a gift from Dustin Ernst and was purified similarly from E. coli strain BL21-AI containing pET14b-*cdsH,* as previously described (67).

### CdsH assay.

2AA deaminase activity was determined using a coupled assay with purified cysteine desulfhydrase (CsdH), RidA, and Y3551, as previously described (67, 69). Assay mixtures (100 μl) contained 100 mM Tris-HCl (pH 8), NADH (250 μM), 5′-phosphate (PLP)-phosphate (30 μM), lactate dehydrogenase (5 U), and purified CdsH (0.27 μM). The reaction mixtures contained RidA or Y3551 (0.19 μM) or an equal volume buffer as a control. The reactions were initiated in triplicate with the addition of freshly prepared l-cysteine (final concentration, 0.5 to 2.5 mM). The reactions were monitored continuously in a 96-well quartz plate by measuring the absorbance at 340 nm for 2 min using a SpectraMax M2 microplate reader. The initial rate of pyruvate formation was calculated from the rate of NADH oxidation in the first 30 s, along with the molar extinction coefficient (ε = 6,200 M^−1 ^cm^−1^).

### *In silico* analyses.

*In silico* predictions of genomic island genetic organization and prediction of promoters were achieved using FGENESB and BPROM (Softberry, Inc., Mt. Kisco, NY) freeware. Alignment of amino acid sequences of Rid proteins was accomplished using the EMBL-MUSCLE tool (https://www.ebi.ac.uk/Tools/msa/muscle/). The protein secondary structure prediction and structural alignments of Y3555 were made using Phyre^2^ (44).

### Statistical analysis.

Statistical analysis was accomplished using GraphPad Prism 7.0, the details for which are provided in “Growth quantification of S. enterica strains,” above, or the legends to Fig. 1 to 3, 6, and 7.

## Supplementary Material

Supplemental file 1
