# Supplementary material for: Putative Horizontally Acquired Genes, Highly Transcribed during Yersinia pestis Flea Infection, Are Induced by Hyperosmotic Stress and Function in Aromatic Amino Acid Metabolism
Source: J Bacteriol. 2020 May 11;202(11):e00733-19. doi: 10.1128/JB.00733-19 (PMC7221256; doi:10.1128/JB.00733-19)
Supplement: Supplemental file 1 [file JB.00733-19-s0001.pdf]

SUPPLEMENTAL FILES FOR:

Putative horizontally acquired genes, highly transcribed during *Yersinia pestis* flea infection, are induced by hyperosmotic stress and function in aromatic amino acid metabolism

Luary C. Martínez-Chavarría [1,2], Janelle Sagawa[1], Jessica Irons[3], Angela K. Hinz[1,4], Athena Lemon[1], Telmo Graça[1], Diana M. Downs[3] and Viveka Vadyvaloo[1*]

1. Paul G. Allen School for Global Animal Health, Washington State University, Pullman, Washington, USA

2. Departamento de Patología, Facultad de Medicina Veterinaria y Zootecnia, Universidad Nacional Autónoma de México, Mexico

3. Department of Microbiology, University of Georgia, 120, Cedar Street, Athens, GA 30602-2605, USA.

4. Department of Chemistry and Biochemistry, Gonzaga University, 502 East Boone Avenue, Spokane WA

***Corresponding author:** Viveka Vadyvaloo, Paul G. Allen School for Global Animal Health, Washington State University, Pullman, WA 99164. Tel: 5093356043 Fax: 5093356328 email: vvadyvaloo@wsu.edu

Running title: Putative horizontally acquired genes of *Y. pestis*

Key words: *Yersinia pestis*, flea infection, hyperosmolarity stress

TABLE S1 Bacterial strains and plasmids used in this study

Strain or plasmid	Genotype or description	Reference or source
**Strains**		
<u>*Y. pestis*</u>		
KIM6+	Pgm+, pCD-, pMT1+, pPst+, parental strain	(1)
VV150	KIM6+ derivative, Δ*y3550*	This study
VV151	KIM6+ derivative, Δ*y3555*	This study
VV278	KIM6+ derivative, Δ*y3550*Δ*y3551*	This study
VV279	KIM6+ derivative, Δ*y3555*Δ*y3550*	This study
VV280	KIM6+ derivative, Δ*y3555*Δ*y3554*Δ*y3553*Δ*y355x*Δ*y3551*Δ*y3550* (*'All'* mutant)	This study
VV267	KIM6+ derivative, Δ*y3555*Δ*y3554*Δ*y3553*	(2)
VV670	KIM6+ derivative, WT *glmS-pstS*::*kan*^{R}	This study
<u>*E. coli*</u>		
DH5α	laboratory cloning strain	Invitrogen
DG44	Δ*tyrB* Δ*aspC*	CGSC Strain#: 5802
DM12740	BL21A1 contains pET20b with *S. enterica ridA*	(3)
DM16279	BL21AI contains pET28a::*y3551*	This study
<u>*Salmonella enterica* serovar Typhimurium LT2</u>		
DM15847	wildtype contains pBAD24 (empty vector)	Downs lab collection
DM16509	DM15847 contains pTRc99A	This study

DM16510	DM15847 contains pTRc55	This study
DM16150	DM15847 *aspC789*::Km	Downs lab collection
DM16434	DM16150 contains pTRc99A (empty vector)	This study
DM16435	DM16150 contains pTRc55	This study
DM14828	*S. enterica* wildtype	Downs lab collection
DM16281	DM14828 pET28a::*y3550*	This study
DM16282	DM14828 pET28a::*y3551*	This study
DM14829	DM14828 derived *ridA1*::Tn10(Tc)	(4)
DM16283	DM14829 contains pET28a::*y3550*	This study
DM16284	DM14829 contains pET28a::*y3551*	This study
DM16293	DM14829 contains pET28b (empty vector)	This study
DM16294	DM14829 contains pET28b::*ridA* (K12)	This study
**Plasmids**		
pC26-pac	pSC101 derivative contains promoterless *luxCDABE* reporter, Km^R	(5)
pCS::P55	pCS26-pac derivative containing *55-lux* transcriptional fusion carrying nucleotides -881/+153 of *y3555* gene, Km^R	This study
pCS::P54	pCS26-pac derivative containing *54-lux* transcriptional fusion carrying nucleotides -428/+130 of *y3554* gene , Km^R	This study
pCS::P53	pCS26-pac derivative containing *53-lux* transcriptional fusion carrying nucleotides -445/+116 of *y3553* gene, Km^R	This study
pCS::P55-54	pCS26-pac derivative containing	

	transcriptional fusion carrying nucleotides -881/+1355 of *y3555* gene, Km^R	This study
pCS::P5x	pCS26-pac derivative containing transcriptional fusion carrying nucleotides -915/+54 of *y3551* gene, Km^R	This study
pCS::P50	pCS26-pac derivative containing transcriptional fusion carrying nucleotides -403/+135 of *y3550* gene, Km^R	This study
pCS::P5x-51-50	pCS26-pac derivative containing transcriptional fusion carrying nucleotides -915/+800 of *y3551* gene, Km^R	This study
pKOBEG	λRed and *sacB* plasmid for mutagenesis, Cm^R	(6)
pKD4	pANTsg derivative template plasmid containing the kanamycin cassette for λRed recombination, Ap^R	(7)
pTRc99A	IPTG-inducible strong hybrid *trp*/*lac* promoter containing plasmid, Ap^R	(8)
pCR4-TOPO	Cloning vector, Km^R, Ap^R	Invitrogen
pCR4::3550	pCR4-TOPO derivative expressing y3550, Km^R, Ap^R	This study
pCR4::3555	pCR4-TOPO derivative expressing y3555, Km^R, Ap^R	This study
pUC18R6KminiTn7T	pUC18 derivative plasmid enabling insertion of genes into *attTn7* transposon insertion site located between	

	the *glmS* and *pstS* genes in the chromosome, Ap^{R}	(9)
E5049::*km*^{R}	pUC18R6KminiTn7T plasmid with kanamycin resistance cassette cloned in between transposition mediating sites	This study
pTRc50	pTRc99A derivative contains *y3550* gene	This study
pTRc51	pTRc99A derivative contains *y3551* gene	This study
pTRc55	pTRc99A derivative contains *y3555* gene	This study
pTRc50_55	pTRc99A derivative contains *y3550* and *y3555*	This study
pTRc51_55	pTRc99A derivative contains *y3551* and y3555	This study
pET28a	Protein Expression vector allowing 6xHis tagging of a gene	EMD Biosciences
pET28a::*y3551*	pET28a derivative contains *y3551* gene	This study
pET28a::*y3550*	pET28a derivative contains *y3550* gene	This study
pET28a::*y3555*	pET28a derivative contains *y3555* gene	This study
pFLP3	Source of Flp recombinase, Tc^{R}, Ap^{R}	(9)

The coordinates for the *lux* fusions are indicated with respect to the translational start codon for each gene. Kan^{R}, kanamycin resistance; Ap^{R}, ampicillin resistance; Tc^{R}, tetracycline resistance.

TABLE S2 Oligonucleotides used in this study

Primer	Sequence (5'-3')	RE
For *lux* fusions		
50R-BHI	TAA*GGATCC*ATTGGCATTCCATGCCAC	BamHI
50F-XhoI	TAA*CTCGAG*TTTATGCTGATTATTTTAAAGGAAC	XhoI
5xR-BHI	GAG*GGATCC*GGCCAATAGCGGAAGGTGC	BamHI
5xF-XhoI	AGG*CTCGAG*GAACTTTTATACAATGGAACGTG	XhoI
53R-BHI	AAC*GGATCC*AGGATGATAATCTGAAAATTC	BamHI
53F-XhoI	TGC*CTCGAG*CTGTAGTAGTCGACTGGCTC	XhoI
54R-BHI	AGC*GGATCC*AATGCGGTCATGTCAAAG	BamHI
54F-XhoI	AAT*CTCGAG*CGTTCTCTTTGGCTGTACCG	XhoI
55R-BHI	CGG*GGATCC*AAGGATCTGCCCAGCACAAC	BamHI
55F-XhoI	GAA*CTCGAG*TGATTCATGATTAACCGAAATGC	XhoI
For RT-PCR analysis		
5051RTR (B_{rt})	CATCAGCAGGCGTGGCCC	
Y3555Set2Fw (A_{rt})	AAATACCTCTTCTCAGGCGGGCAT	
Gene deletion		
3550-H1P1	CAATAATGGAGAGTTATCATGAATAGACGTGTAATCAAT	
	CCCTGTGTAGGCTGGAGCTGCTTCG	
3550-H2P2	CTACTCTTCATTTTATTATCAGCCCAAGCAAGCAGTCGC	
	TTCCATATGAATATCCTCCTTAGTTC	
3555-H1P1	TTATTAAAGGGGAATACCATGAATACGATTAGAAGAAAA	

	ATGTGTGTAGGCTGGAGCTGCTTCG	
3555-H2P2	CGCTAACTGCATTAATGCCTGTTGGATTCGAGTCAAAAC	
	TGCCATATGAATATCCTCCTTAGTTC	
3551-H1P1	TAAAATAGAGGATATATCATGAAGTCAGTCATTGAAACA	
	AAATGTAGGCTGGAGCTGCTTC	
For cloning		
p270	AC*GGATCC*ATGAATAGACGTGTAATCAATCCCG	
p271	CA*CTCGAG*TCAGCCCAAGCAAGCAGTCGC	
p272	AC*GGATCC*ATGAAGTCAGTCATTGAAACAAAAAATGC	
p273	CA*CTCGAG*TCAAACCCCAGCAATAACTTCTATTTC[1]	
p274	AC*GGATCC*ATGAATACGATTAGAAGAAAAATGCGTC	
p275	CA*CTCGAG*TTATCCATGGCAGATCCCCTTTTCC	
p508	CGATAAGCTTGATTTAGTAAGCCTGTGATTTGC	
p509	TGTGAAGCTTATCCATGGCAGATCCCCTTTTCC	
p395/pKD4KanF	ATTAGGATCCGAACACGTAGAAAGCCAGTCC	
p396/pKD4KanR	CAGCGAATTCTCAGAAGAACTCGTCAAGAAGGC	
pstSup2	GCTATACGTGTTTGCTGATCAAGATGC	(9)
pTn7R	CACAGCATAACTGGACTGATTTC	(9)
3550-R	CGC CCA TTT AAT GAT GAG TGC	
3550-F	TCC CAC TGC TGA TTA GTA TCG	
3555-R	CAA CAC CGG ACT GAG CGA TC	
3555-F	ATT TTG TGT CAA CTG ACT GAT TG	
For qPCR		
y3550Set1Fw	GCATGGAATGCCAATGGCGAGTTA	

y3550Set1Rev	TAGACGTACAACATCAGCAGGCGT
y3551Set1Fw	TGCTTCAGGGCAGATACCGATCAA
y3551Set1Rev	GTTCCAGCCTGTAGAAGAACAGCA
y3553Set1Fw	TCATCCTGGTGCTGTTCGTAGT
y3553Set1Rev	TCCATCAGACATTTCAACTCGCCG
y3554Set3Fw	GCATCATTGGTATTCTGCGTGCCA
y3554Set3Rev	CGCCAACTAATGCGGTTGCGATAA
y3555Set2Fw	AAATACCTCTTCTCAGGCGGGCAT
y3555Set2Rev	ATGAGAGGAACCCACTTTCAGGCA
gyrBFw	TCGCCGTGAAGGTAAAGTTC
gyrBRv	TTTGCTCAGTTTCACCCACC

Protein %ID

1: Y3555	100.00	20.17	19.88	15.80	18.97
2: Y2760_AspC	20.17	100.00	85.86	41.41	42.42
3: Se_AspC	19.88	85.86	100.00	42.17	42.42
4: Y0579_TyrB	15.80	41.41	42.17	100.00	72.80
5: Se_TyrB	18.97	42.42	42.42	72.80	100.00

```
Y3555         1 -----MNTIRRKMRPEFQGLQGGLFSSVKKADVGTAVLD----------LMANGVDM--L
Y2760_AspC    1 MEASDMF-------------------EKIT-AAPADPILG-LTDIFRADDRAHK-INLGIG
Y0579_TyrB    1 -----MF-------------------QNVD-AYAGDPILS-LMESFKADNRAHK-VNLSIG
STM0998_AspC  1 -----MF-------------------ENIT-AAPADPILG-LADLFRADDRPGK-INLGIG
STM4248_TyrB  1 -----MF-------------------QKVD-AYAGDPILS-LMERFKDDSRHDK-VNLSIG

Y3555        44 CWADPFFPDPVLP--EHISQAVIKSME--NGSASHYTMPIGNPELKEKIALKLQRYNNLT
Y2760_AspC   40 VYKDETGKTPVLT---SVKKAEQYLLE-N-EATKNYLGIDGLPVFASCTQELLFGANSAI
Y0579_TyrB   35 LYYNEQGEIPQMQ---AVDAAEAQLSTQP-HGTPVYLPMEGLQSYRTAIQQLLFGHDHPM
STM0998_AspC 35 VYKDETGKTPVLT---SVKKAEQYLLE-N-ETTKNYLGIDGIPEFARCTQELLFGKGSAL
STM4248_TyrB 35 LYYNEDGIIPQLK---TVAEAEARLNAQP-HGASLYLPMEGLNTYRHTIAPLLFGADHPV

Y3555       100 VEAQR-NILITPGSDSGLLFAMMPFIN---NDDEVLIHSPSYPSNFLNVELLGGKPISVE
Y2760_AspC   95 IADKRARTAQTPGGTGGLRIAA-DFIAHQTSAKRVWVSNPSWPNHKNVFEAAGLEVVEYA
Y0579_TyrB   91 LVQQRVATIQTVGGSGALKVGA-DFLNHYFPDSQVWVSDPTWENHVAIFSGAGFKVNTYP
STM0998_AspC 90 INDKRARTAQTPGGTGALRIAA-DFLAKNTPVKRVWVSNPSWPNHKSVFNAAGLEVREYA
STM4248_TyrB 91 LQQQRVATIQTLGGSGALKVGA-DFLKRYFPDAGVWVSDPTWENHIAIFAGAGFEVSTYP

Y3555       156 LKAENNFQIDIKDFENKITEK-TKMVILTN--PNNPTGTVLRRESLQAIADFIIA-----
Y2760_AspC  154 YYDAANHALDFDGLLNSLSEAQAGDVVLFHGCCHNPTGIDPTETQWSQLAELSVA-----
Y0579_TyrB  150 YFDNDKLAVKFDPMLATLQQLPARSIVLLHPCCHNPTGSDLTNAQWDRLIDVVKE-----
STM0998_AspC149 YYDAENHTLDFEALQASLSEAQAGDVVLFHGCCHNPTGIDPTLEQWQVLAELSVE-----
STM4248_TyrB150 WYDDATNGIRFNDLLATLNTLPARSIVLLHPCCHNPTGADLTPSQWDAVIEIVKA-----

Y3555       208 -HDLILVVDQAFE---DAIFDEIEFISIASLPGMWERTVSVFSFSKGMGLSGFRVGYLVA
Y2760_AspC  209 -KGWLPLFDFAYQGFANGLEEDAQG--LRIFAATHQELIVCSSYSKNFGLYNERVGACTL
Y0579_TyrB  205 -RELIPFLDIAYQGFGAGLNEDAYA--IRAMAAVGLPCLISNSFSKIFSLYNERVGGLSV
STM0998_AspC204 -KGWLPLFDFAYQGFARGLEEDAEG--LRAFAALHKELIVASSYSKNFGLYNERVGACTL
STM4248_TyrB205 -RDLIPFLDIAYQGFGAGMDDDAYV--IRAIASAGLPALVSNSFSKIFSLYGERVGGLSV
                                     ★

Y3555       264 ---DAQIVDVLFGCTVNVVGATNTS---SQAGMIAAL-DEPSFMGEYTQIFER-------
Y2760_AspC  266 VAADSNVADTAFSQVKAVIRANYSNPPAHGASVVATILSNAALRAIWEQELTDMRQRIQR
Y0579_TyrB  262 VCESDEAAGRVLGQLKATVRRNYSSPPNFGAQVVSKVLNDTVLRAQWQAEVEQMRLRILD
STM0998_AspC261 VAADAETVDRAFSQMKSAIRANYSNPPAHGASIVATILSNDALRAIWEQELTDMRQRIQR
STM4248_TyrB262 VCEDAEIAARVLGQLKATVRRIYSSPPCFGAQVVATVLGDEALKAGWLAEVDAMRNRIIS

Y3555       310 RRKVVFEMMNAIPGVCMVMPESGFLSWIDISKLGTSTFICDYLLQHAHVMVNSGVPYGQG
Y2760_AspC  326 MRQLFVNTLQ------EKGAQQDF----------------SFIINQNGMFSFSGLTKEQV
Y0579_TyrB  322 MRHTLVEALK------ASLPERNF----------------DYLLQQRGMFSYTGFSEAQV
STM0998_AspC321 MRQLFVNTLQ------EKGANRDF----------------SFIIKQNGMFSFSGLTKDQV
STM4248_TyrB322 MRQTLVKELK------AEMPDRNF----------------DYLLQQRGMFSYTGLSEEQV
```

```
Y3555        370 GEGYIRLVHGCYKDDEKLYAVLT-RIQQALMQLAKEKGICHG------------------
Y2760_AspC   364 ----LR-----LRDEFAVYAVNSGRVNVAGMTPDNMAPLCEA------------------
Y0579_TyrB   360 ----ES-----LREEFGVYLIASGRMCMAGVNHQNVEQVAVA------------------
STM0998_AspC359 ----LR-----LREEFGVYAVASGRVNVAGMTPDNMAPLCEA------------------
STM4248_TyrB360 ----DR-----LRDEFGVYLIASGRMCVAGLNASNVHRVAKA------------------


Y3555            -----
Y2760_AspC   397 IVAVL
Y0579_TyrB   393 FAAVQ
STM0998_AspC392 IVAVL
STM4248_TyrB393 FAAVM
```

<u>Figure S1</u>. Alignments of the Y3555 and *Y. pestis* KIM6+ and *S. enterica* AspC and TyrB. *denotes the glutamate instead of aspartate that is predicted to coordinate the pyridine nitrogen of PLP in the active site

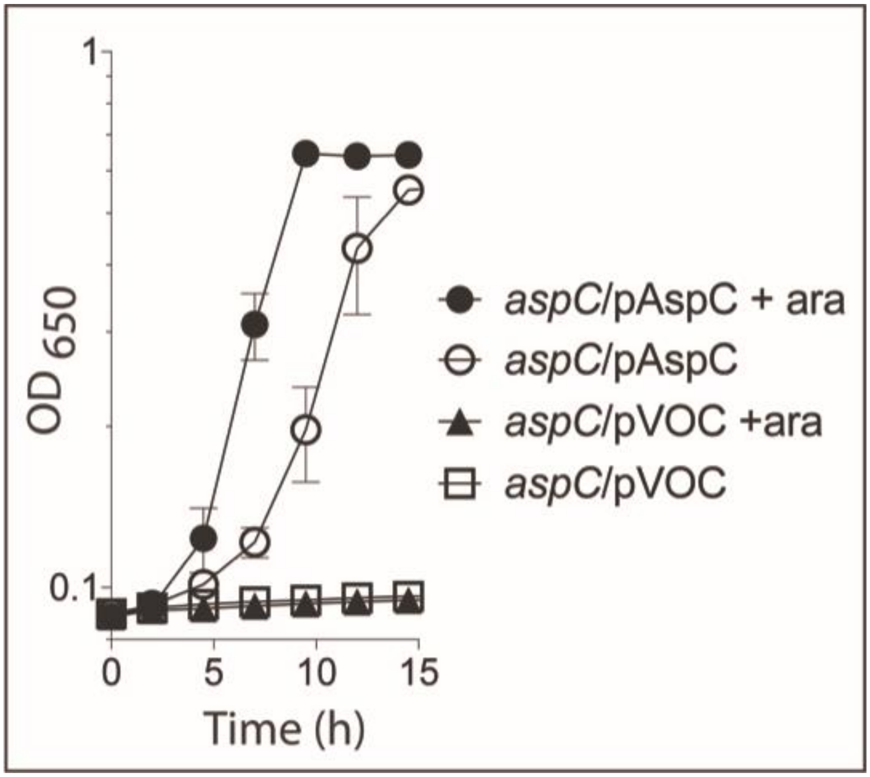

<u>Figure S2</u>. *S. enterica aspC* expressed *in trans* complements growth defects of an *S. enterica aspC* mutant The *S. enterica aspC789*::km mutant (DM16150) with either a pBAD24 empty vector control (*aspC*/pVOC), or a pBAD24 plasmid expressing *S. enterica aspC* (*aspC*/pAspC) were grown in minimal glucose (11 mM) medium without, or with 0.2% arabinose (+ara) as indicated in legend. Error bars represent mean(±SEM) from three biological replicates.

Protein %ID

1: Y3555	100.00	86.59	86.59	86.59	86.83	19.60	19.03	16.43	18.70
2: 07704	86.59	100.00	98.05	98.05	98.29	19.03	18.47	16.71	17.85
3: 072382	86.59	98.05	100.00	100.00	99.76	19.60	18.75	15.86	17.85
4: 093506	86.59	98.05	100.00	100.00	99.76	19.60	18.75	15.86	17.85
5: Se_AspB	86.83	98.29	99.76	99.76	100.00	19.60	18.75	15.86	17.85
6: Y2760_AspC	19.60	19.03	19.60	19.60	19.60	100.00	85.86	41.41	42.42
7: Se_AspC	19.03	18.47	18.75	18.75	18.75	85.86	100.00	42.17	42.42
8: Y0579_TyrB	16.43	16.71	15.86	15.86	15.86	41.41	42.17	100.00	72.80
9: Se_TyrB	18.70	17.85	17.85	17.85	17.85	42.42	42.42	72.80	100.00

```
Y3555          1 MNTIRRKMRPEFQGLQGGLFSSVKKADVGTAVLDLMANGVDM-------LCWADPFFPDP
Y2760_AspC     1 MEASD-------------MFEKIT-AAPADPILGLTDIFRADDRAHKINLGIGVYKDETG
Y0579_TyrB     1 M-----------------FQNVD-AYAGDPILSLMESFKADNRAHKVNLSIGLYYNEQG
STM0998_AspC   1 M-----------------FENIT-AAPADPILGLADLFRADDRPGKINLGIGVYKDETG
STM4248_TyrB   1 M-----------------FQKVD-AYAGDPILSLMERFKDDSRHDKVNLSIGLYYNEDG
07704          1 MSTIRRKMRPVFQGLQGGLFNSVKKADVGTAVLELMANGVDM-------LCWADPFFPDS
072382         1 MSTIRRKMRPVFQGLQGGLFNSVKKADVGTAVLELMANGVDM-------LCWADPFFPDS
093506         1 MSTIRRKMRPVFQGLQGGLFNSVKKADVGTAVLELMANGVDM-------LCWADPFFPDS
Se_AspB        1 MSTIRRKMRPVFQGLQGGLFNSVKKADVGTAVLELMANGVDM-------LCWADPFFPDS


Y3555         54 VLPE--HISQAVIKSM-ENGSASHYTMPIGNPELKEKIALKLQRYNNLTVEAQRNIL-IT
Y2760_AspC    47 KTPVLTSVKKAEQYLL-ENEATKNYLGIDGLPVFASCTQELLFGANSAIIADKRARTAQT
Y0579_TyrB    42 EIPQMQAVDAAEAQLSTQPHGTPVYLPMEGLQSYRTAIQQLLFGHDHPMLVQQRVATIQT
STM0998_AspC  42 KTPVLTSVKKAEQYLL-ENETTKNYLGIDGIPEFARCTQELLFGKGSALINDKRARTAQT
STM4248_TyrB  42 IIPQLKTVAEAEARLNAQPHGASLYLPMEGLNTYRHTIAPLLFGADHPVLQQQRVATIQT
07704         54 VLPE--HISQAVINSM-KNGNASHYTMPIGNPDLKEKIALKLLRKNNISVDAQRNIL-IT
072382        54 VLPE--HISQAVIKSM-ENGNASHYTMPIGNPDLKEKIAIKLLRKNNISVDAQRNIL-IT
093506        54 VLPE--HISQAVIKSM-ENGNASHYTMPIGNPDLKEKIAIKLLRKNNISVDAQRNIL-IT
Se_AspB       54 VLPE--HISQAVIKSM-ENGNASHYTMPIGNPDLKEKIALKLLRKNNISVDAQRNIL-IT


Y3555        110 PGSDSGLLFAMMPFINN---DDEVLIHSPSYPSNFLNVELLGGKPISVELKAENNFQIDI
Y2760_AspC   106 PGGT-GGLRIAADFIAHQTSAKRVWVSNPSWPNHKNVFEAAGLEVVEYAYYDAANHALDF
Y0579_TyrB   102 VGGS-GALKVGADFLNHYFPDSQVWVSDPTWENHVAIFSGAGFKVNTYPYFDNDKLAVKF
STM0998_AspC101 PGGT-GALRIAADFLAKNTPVKRVWVSNPSWPNHKSVFNAAGLEVREYAYYDAENHTLDF
STM4248_TyrB102 LGGS-GALKVGADFLKRYFPDAGVWVSDPTWENHIAIFAGAGFEVSTYPWYDDATNGIRF
07704        110 PGSDSGLLFAMMPFIED---GDEVIIHSPSYPSNFLNVELLGGTPVSVELKAENNFQIEI
072382       110 PGSDSGLLFAMMPFIED---GDEVIIHSPSYPSNFLNVELLGGTPVSVELKVENNFQIEI
093506       110 PGSDSGLLFAMMPFIED---GDEVIIHSPSYPSNFLNVELLGGTPVSVELKVENNFQIEI
Se_AspB      110 PGSDSGLLFAMMPFIED---GDEVIIHSPSYPSNFLNVELLGGTPVSVELKVENNFQIEI


Y3555        167 KDFENKITEK-TKMVILTN--PNNPTGTVLRRESLQAIADFIIAHDLILVVDQAFED---
Y2760_AspC   165 DGLLNSLSEAQAGDVVLFHGCCHNPTGIDPTETQWSQLAELSVAKGWLPLFDFAYQGFAN
Y0579_TyrB   161 DPMLATLQQLPARSIVLLHPCCHNPTGSDLTNAQWDRLIDVVKERELIPFLDIAYQGFGA
STM0998_AspC160 EALQASLSEAQAGDVVLFHGCCHNPTGIDPTLEQWQVLAELSVEKGWLPLFDFAYQGFAR
STM4248_TyrB161 NDLLATLNTLPARSIVLLHPCCHNPTGADLTPSQWDAVIEIVKARDLIPFLDIAYQGFGA
07704        167 SDFEEKITEK-TKMVILTN--PNNPTGTVLRRESLQALADFVIEHDLVLVVDQAFED---
072382       167 SDFEEKITEK-TKMVILTN--PNNPTGTVLRRESLQALADFVIDHDLVLVVDQAFED---
093506       167 SDFEEKITEK-TKMVILTN--PNNPTGTVLRRESLQALADFVIDHDLVLVVDQAFED---
```

```
Se_AspB      167 SDFEEKITEK-TKMVILTN--PNNPTGTVLRRESLQALADFVIDHDLVLVVDQAFED---

Y3555        221 AIFDEIEFISIASLPGMWERTVSVFSFSKGMGLSGFRVGYL---VADAQIVDVLFGCTVN
Y2760_AspC   225 GLEEDAQG--LRIFAATHQELIVCSSYSKNFGLYNERVGACTLVAADSNVADTAFSQVKA
Y0579_TyrB   221 GLNEDAYA--IRAMAAVGLPCLISNSFSKIFSLYNERVGGLSVVCESDEAAGRVLGQLKA
STM0998_AspC220 GLEEDAEG--LRAFAALHKELIVASSYSKNFGLYNERVGACTLVAADAETVDRAFSQMKS
STM4248_TyrB221 GMDDDAYV--IRAIASAGLPALVSNSFSKIFSLYGERVGGLSVVCEDAEIAARVLGQLKA
07704        221 AIFDNIEFISIASLPGMWERTISVFSFSKGMGLSGFRVGYL---VADDHIMDALYGCTVN
072382       221 AIFDNIEFISIASLPGMWERTISVFSFSKGMGLSGFRVGYL---VADDHIMDALYGCTVN
093506       221 AIFDNIEFISIASLPGMWERTISVFSFSKGMGLSGFRVGYL---VADDHIMDALYGCTVN
Se_AspB      221 AIFDNIEFISIASLPGMWERTISVFSFSKGMGLSGFRVGYL---VADDHIMDALYGCTVN
                     ★

Y3555        278 VVGATNTSSQ---AGM---------IAALDEPSFMGEYTQIFERRRKVVFEMMN--AIPG
Y2760_AspC   283 VIRANYSNPPAHGASVVATILSNAALRAIWEQELTDMRQRIQRMRQLFVNTLQEKGAQQD
Y0579_TyrB   279 TVRRNYSSPPNFGAQVVSKVLNDTVLRAQWQAEVEQMRLRILDMRHTLVEALKASLPERN
STM0998_AspC278 AIRANYSNPPAHGASIVATILSNDALRAIWEQELTDMRQRIQRMRQLFVNTLQEKGANRD
STM4248_TyrB279 TVRRIYSSPPCFGAQVVATVLGDEALKAGWLAEVDAMRNRIISMRQTLVKELKAEMPDRN
07704        278 VVGATNTSSQ---AGM---------IAALNDPSFIDTYNQIFEHRRKVVFEMMN--AIPG
072382       278 VVGATNTSSQ---AGM---------IAALNEPSFIDTYNQIFDHRRKVVFEMMN--AIPG
093506       278 VVGATNTSSQ---AGM---------IAALNEPSFIDTYNQIFDHRRKVVFEMMN--AIPG
Se_AspB      278 VVGATNTSSQ---AGM---------IAALNEPSFIDTYNQIFDHRRKVVFEMMN--AIPG

Y3555        324 VCMVMPESGFLSWIDISKLGTSTFICDYLLQHAHVMVNSGVPYGQGGEGYIRLVHGCYKD
Y2760_AspC   343 FSFIINQNGMFSFSGLTKEQVLRL----RDEFAVYAVNSGR---------VN-VAGMTPD
Y0579_TyrB   339 FDYLLQQRGMFSYTGFSEAQVESL----REEFGVYLIASGR---------MC-MAGVNHQ
Se_AspC      338 FSFIIKQNGMFSFSGLTKDQVLRL----REEFGVYAVASGR---------VN-VAGMTPD
Se_TyrB      339 FDYLLQQRGMFSYTGLSEEQVDRL----RDEFGVYLIASGR---------MC-VAGLNAS
07704        324 VSMVMPESGFLSWIDISKLGSSAFICDYLLEHAQVVVNSGVPYGEGGEGYIRLVHGCYKD
072382       324 VSMVMPESGFLSWIDISKLGSSAFICDYLLEHAQVVVNSGVPYGEGGEGYIRLVHGCYKD
093506       324 VSMVMPESGFLSWIDISKLGSSAFICDYLLEHAQVVVNSGVPYGEGGEGYIRLVHGCYKD
Se_AspB      324 VSMVMPESGFLSWIDISKLGSSAFICDYLLEHAQVVVNSGVPYGEGGEGYIRLVHGCYKD

Y3555        384 D-EKLYAVLTRIQQALMQLAKEKGICHG
Y2760_AspC   389 NMAPLCEAIVAVL---------------
Y0579_TyrB   385 NVEQVAVAFAAVQ---------------
Se_AspC      384 NMAPLCEAIVAVL---------------
Se_TyrB      385 NVHRVAKAFAAVM---------------
07704        384 D-EKLFAVLTRIQSALTMLAKEKGLDHE
072382       384 D-EKLCAVLTRIQSALTMLAKEKGLDHE
093506       384 D-EKLCAVLTRIQSALTMLAKEKGLDHE
Se_AspB      384 D-EKLCAVLTRIQSALTMLAKEKGLDHE
```

Figure S3. Alignments of Y3555 and 07704, 072382, 093506, AspB from CDC isolates of *S. enterica*. *denotes the glutamate instead of aspartate that is predicted to coordinate the pyridine nitrogen of PLP in the active site.

SUPPLEMENTAL REFERENCES

1. **Une T, Brubaker RR.** 1984. *In vivo* comparison of avirulent Vwa- and Pgm- or Pstr phenotypes of yersiniae. Infect Immun **43:**895-900.
2. **Fukuto HS, Svetlanov A, Palmer LE, Karzai AW, Bliska JB.** 2010. Global gene expression profiling of Yersinia pestis replicating inside macrophages reveals the roles of a putative stress-induced operon in regulating type III secretion and intracellular cell division. Infect Immun **78:**3700-3715.
3. **Lambrecht JA, Browne BA, Downs DM.** 2010. Members of the YjgF/YER057c/UK114 family of proteins inhibit phosphoribosylamine synthesis in vitro. J Biol Chem **285:**34401-34407.
4. **Ernst DC, Lambrecht JA, Schomer RA, Downs DM.** 2014. Endogenous synthesis of 2-aminoacrylate contributes to cysteine sensitivity in *Salmonella enterica*. J Bacteriol **196:**3335-3342.
5. **Bjarnason J, Southward CM, Surette MG.** 2003. Genomic profiling of iron-responsive genes in *Salmonella enterica* serovar *typhimurium* by high-throughput screening of a random promoter library. J Bacteriol **185:**4973-4982.
6. **Derbise A, Lesic B, Dacheux D, Ghigo JM, Carniel E.** 2003. A rapid and simple method for inactivating chromosomal genes in *Yersinia*. FEMS Immunology & Medical Microbiology **38:**113-116.
7. **Datsenko KA, Wanner BL.** 2000. One-step inactivation of chromosomal genes in Escherichia coli K-12 using PCR products. Proc Natl Acad Sci U S A **97:**6640-6645.
8. **Amann E, Ochs B, Abel KJ.** 1988. Tightly regulated tac promoter vectors useful for the expression of unfused and fused proteins in Escherichia coli. Gene **69:**301-315.
9. **Choi KH, Gaynor JB, White KG, Lopez C, Bosio CM, Karkhoff-Schweizer RR, Schweizer HP.** 2005. A Tn7-based broad-range bacterial cloning and expression system. Nat Methods **2:**443-448.

1
